# Prevalence and Epidemiological Characteristics of Anogenital Warts Among Recently Diagnosed HIV-Positive Women on Antiretroviral Therapy in Lagos, Nigeria

**DOI:** 10.7759/cureus.56251

**Published:** 2024-03-16

**Authors:** Ayodeji K Adefemi, Adeyemi A Okunowo, Rose I Anorlu

**Affiliations:** 1 Department of Obstetrics and Gynaecology, Lagos State University Teaching Hospital, Lagos, NGA; 2 Department of Obstetrics and Gynaecology, College of Medicine, University of Lagos, Lagos, NGA; 3 Department of Obstetrics and Gynaecology, Lagos University Teaching Hospital, Lagos, NGA

**Keywords:** people living with hiv/aids, nigeria, lagos, prevalence, epidemiology, anti-retroviral therapy, human papillomavirus (hpv), hiv-positive women, human immunodeficiency virus (hiv), anogenital warts

## Abstract

Background

Anogenital warts (AGWs) are a prevalent condition resulting from human papillomavirus (HPV) infection, which is the most frequently encountered sexually transmitted infection (STI) on a global scale. Women who are HIV-positive experience a disproportionately high burden of AGWs compared to other populations. It is imperative to comprehend the epidemiological factors linked to the disease within this particular at-risk population.

Objectives

The objective of the study was to ascertain the prevalence of AGWs and its demographic and socio-biological epidemiological features among recently diagnosed HIV-positive women (HPW) in Lagos, Nigeria.

Materials and methods

The research was a descriptive cross-sectional study conducted among a sample of 420 recently diagnosed HPW. The study was conducted at the HIV clinic of a tertiary health institution located in Lagos, Nigeria. The participants clinically diagnosed with AGWs were classified as the study group, while individuals without AGWs were classified as the comparison group. Interviewer-administered pretested questionnaires were utilized to gather pertinent demographic and socio-biological epidemiological data from the participants involved in the study. The data were analyzed using IBM SPSS Statistics for Windows, Version 23.0 (Released 2015; IBM Corp., Armonk, New York, USA).

Results

The prevalence of AGWs among recently diagnosed HPW was found to be 8.5% (34/402). These warts were frequently observed on the vulvar labia (35.3%, 12/34), vaginal walls (14.7%, 5/34), and perianal region (14.7%, 5/34). It is worth noting that over a third of cases (35.3%, 12/34) involved multiple areas within the anogenital region. The diagnosis of AGWs was found to have significant associations with occupation (p=0.005), marital status (p<0.001), and educational status (p=0.028). The majority of HPW diagnosed with AGWs were unemployed (32.4%, 11/34), single (47.1%, 16/34), and did not have tertiary education (94.1%, 32/34). The utilization of oral contraceptive pills (OCPs), smoking, low CD4 count, and high viral load were the significant socio-biological factors associated with the diagnosis of AGWs (p<0.001, respectively).

Conclusion

The study found that the prevalence of AGW among HPW was 8.5% (34/402). Several epidemiological factors, including occupation, marital status, education, CD4 count, viral load, history of OCP use, and smoking, were found to be significantly associated with the diagnosis of AGW. There is a need to conduct more comprehensive studies to thoroughly assess the impact of these epidemiological factors.

## Introduction

Anogenital warts (AGWs) are a result of infection with human papillomavirus (HPV), which is the most prevalent sexually transmitted infection globally [1]. A significant number of sexually active individuals, both male and female, are likely to get the HPV infection at some point in their lives. Nevertheless, this condition typically resolves on its own, and the virus is naturally eliminated by the immune-competent human system [2,3]. In cases where the human immune system is compromised or defective, such as in individuals who are HIV positive, the virus can persist within the body and lead to a range of diseases associated with HPV [1,3,4].

AGW is a sexually transmitted disease primarily caused by low-risk HPV (LrHPV) types 6 and 11, which account for over 90% of cases [1,5,6]. HPV is epitheliotrophic in nature, and it specifically targets and infects the epithelial surfaces, leading to the development of aberrant growth. This growth might manifest as cutaneous, affecting the skin surfaces, or non-cutaneous, affecting the mucosa surfaces [5]. AGWs frequently manifest in the vulvar, vaginal, cervical, perineum, and perianal areas in females [7,8]. In males, it can manifest in various areas, including the penile shaft, glans, external urethral meatus, scrotum, urethra, perineum, and anal regions [7]. AGWs can manifest as discrete or confluent features, displaying variable sizes. These lesions typically appear as reddish, pinkish, or brownish hyperpigmented areas, which may have a fleshy, papular, keratotic, or cauliflower-like texture [7-10].

The majority of AGWs are typically asymptomatic and are commonly detected incidentally, either by the patient or by the attending physician during clinical examination [9]. Nevertheless, it may be linked to various issues, including physical, emotional, psychological, and economic challenges. Typical physical symptoms commonly associated with AGW include pruritus, discomfort, vaginal discharge, bleeding, burning sensations, and dyspareunia [7-9]. Emotional and psychological challenges related to AGW encompass various psychosocial and psychosexual issues. Psychosocial problems may manifest as feelings of shame, embarrassment, guilt, and depression. On the other hand, psychosexual problems may involve a decrease in sexual drive due to the fear of one's partner discovering their AGW condition, experiencing embarrassment after disclosure, and concerns about transmitting AGW to sexual partners [6,8]. Additionally, it is important to note that this condition carries a substantial economic burden, particularly among individuals in the young reproductive-aged demographic who may lack the financial means to cover the extensive costs associated with both direct and indirect treatment expenses [6,9].

The correlation between HIV and HPV remains incompletely elucidated; nonetheless, it is postulated that HIV may exhibit a symbiotic association with HPV infection. Research has demonstrated that the presence of HPV infection significantly elevates the likelihood of acquiring HIV infection. Conversely, the presence of HIV infection intensifies the persistence of HPV infection and its progression to disease [11-14]. The interaction between these factors contributes to the increased prevalence of HPV infection and the significant burden of HPV-related diseases observed in people living with HIV and AIDS (PLWHA) [15]. It is thus unsurprising that AGW, the most prevalent disease caused by HPV, exhibits a higher prevalence and severity among PLWHA in comparison with individuals without HIV/AIDS [13].

The sub-Saharan Africa region exhibits a notable prevalence of HPV and HIV infections, which plays a significant role in the heightened burden of AGW and its unfavorable outcomes in the region [13,14,16]. Furthermore, several factors have been identified as potential influencers of the burden of AGW among PLWHA. These factors include the utilization and adherence to antiretroviral therapy (ART), the duration of ART treatment, the level of HIV-related immunosuppression, the CD4+ cell count, and the HIV viral load [13,17]. Nevertheless, the epidemiological implications of certain factors remain uncertain, particularly within our specific context. Our study aimed to assess the prevalence of AGW and its demographic and socio-biological epidemiological features among recently diagnosed HIV-positive women (HPW) in Lagos, Nigeria.

## Materials and methods

Study design and setting

The research was a comparative cross-sectional study carried out among recently diagnosed HPW who attended the AIDS Prevention Initiative in Nigeria (APIN) clinic at Lagos University Teaching Hospital, Lagos, Nigeria, between March 2016 and September 2016. The APIN clinic provides healthcare services to a patient population of over 10,000 PLWHA, with females comprising more than 60% of the total. The clinic operates a general outpatient clinic daily, while specialized clinics are scheduled on specific days. HPW were provided with counseling on cervical cancer screening and were referred to the cytology and colposcopy clinic for screening. Individuals experiencing gynecological issues received counseling and treatment and were referred to the gynecological outpatient clinic for further assessment and treatment.

Study population and eligibility criteria

The study population consisted of recently diagnosed HPW who sought treatment at the APIN clinic within the institution. In the context of this study, a recently diagnosed individual with HIV/AIDS refers to someone who has received a diagnosis of HIV/AIDS within the past six months and has recently initiated ART. Patients with clinical evidence of AGWs on pelvic examination were classified as the study group, while those without clinical evidence of AGWs were classified as the comparison group. 

The study included recently diagnosed HPW, aged 18 years and older, who were sexually active, and had attended the APIN clinic. Only those who provided informed consent were included in the study. In contrast, individuals who were under the age of 18, not sexually active, or unwilling to participate in the study were excluded from the research.

Sample size determination and recruitment of study participants

The sample size was determined using the formula (n=Z^2^p (1−p)/d^2^) [18], with an absolute error margin of 5% (d=0.05), type 1 error of 5% (Z=1.96), and a prevalence of AGW (p) of 9.3% [19]. The minimum sample size was determined to be 124, taking into account a non-response rate of 15%. HPW who met the eligibility criteria were provided with information about the study's purpose and subsequently recruited using a convenient sampling method following the acquisition of informed consent.

Instrument of survey and data collection

The survey instrument utilized in this study was a pretested structured data form designed to collect pertinent demographic and socio-biological information from the participants. The demographic information collected included the participants' age, religion, occupation, educational status, and marital status. The socio-biological data collected included information on the type of marriage, usage and duration of OCPs, condom usage, smoking history, and alcohol consumption. Additional socio-biological data collected included the number of childbirths, total count of lifetime sexual partners, history of multiple sexual partners in the spouse, and details regarding the viral load and CD4 count levels.

The questionnaires were administered by trained research assistants to all eligible study participants in an interviewer-administered format following the acquisition of informed consent. Before the study began, a pretest of the questionnaire was conducted among a convenient sample of 20 recently diagnosed HPW who were attending the HIV clinic at another hospital. The purpose of this pretest was to evaluate the clarity of the questionnaire's instructions, the ease of comprehension, the appropriateness of its content, and to identify any potential ambiguity within the questionnaire. The findings from the pilot study were used to make appropriate enhancements to the study questionnaire.

Clinical examination

All participants underwent a comprehensive examination of their external genitalia conducted by the attending gynecologist. The clinical examination took place in a designated consultation room with a female chaperone following the acquisition of informed consent. The participants were positioned supine on the examination couch, with the vulva appropriately exposed. A comprehensive examination of the entire vulva, perineum, and anal regions was performed under proper illumination to assess for the presence of warts. A speculum of suitable size was inserted to expose the vaginal walls and cervix adequately. Subsequently, a thorough examination was conducted to determine the presence of any warts. HPW with identifiable AGW were counseled and administered suitable treatment.

Data analysis

The data were entered into the computer, cleaned, and validated. Data analysis was done using IBM SPSS Statistics for Windows, Version 23.0 (Released 2015; IBM Corp., Armonk, New York, USA). Descriptive statistics were presented as frequencies and percentages in tables or figures. Continuous data were assessed for normality using Shapiro-Wilk test. Normally distributed variables were presented as mean±standard deviation (SD), while skewed variables were expressed as median and interquartile range (IQR). Data were grouped into categories for ease of analysis, and bivariate analysis was done using Pearson’s chi-square test (or Fisher’s exact test) and Student’s t-test (or Mann-Whitney U test) to compare categorical and continuous variables, respectively. Statistical significance was set at a p-value <0.05 and a 95% confidence interval.

Ethical consideration

Ethical approval (ADM/DCST/HREC/APP/394) was obtained from the Human Research and Ethical Committee of Lagos University Teaching Hospital before conducting the study. Informed consent was obtained from all participants before their enrollment in the study. The study was carried out in accordance with the Declaration of Helsinki (1964).

## Results

Out of a total of 420 participants that were enrolled in the study, 402 (95.7%) had complete data and were included in the final analysis.

Demographic characteristics of study participants

Table 1 presents the characteristics of the study participants. The mean age of the participants was 30.7±7.6 years, with the majority (43.3%, 174/402) between the ages of 30 and 39 years followed by 20 and 29 years (40.3%, 162/402). A greater proportion of the participants were married (64.2%, 258/402), Christians (92.8%, 373/402), had a secondary level of education (46.0%, 185/402), and had an unskilled occupation (44.8%, 180/402).

**Table 1:** Demographic characteristics of the study participants. N: total population; SD: standard deviation. The data have been represented as frequency (N), percentage (%), and mean±SD.

Demographic characteristics	Frequency N=402	Percentage (%)
Age groups (in years)		
<20	23	5.7
20–29	162	40.3
30–39	174	43.3
≥40	43	10.7
Mean age±SD	30.7±7.6	
Occupation		
Unemployed	53	13.2
Unskilled labor	180	44.8
Skilled labor	142	35.3
Professionals	27	6.7
Marital status		
Single	115	28.6
Married	258	64.2
Divorced	21	5.2
Widowed	8	2.0
Religion		
Christianity	373	92.8
Islam	27	6.7
Traditional religion	2	0.5
Educational status		
No formal education	62	15.4
Primary education	111	27.6
Secondary education	185	46.0
Tertiary education	44	10.9

Socio-biological characteristics of study participants

The median parity of the participants was 4 (2-5), with the majority having a parity of 2 and above (67.4%, 271/402). All the participants were sexually active, with the majority having between six and 10 lifetime sexual partners (91.3%, 367/402) and a median total lifetime sexual partner of 8 (7-9). Most of the women (96.8%, 389/402) had sexual partners who had other sexual partners and used condom for contraception (87.6%, 352/402). The median CD4 count was 440 (291-593) cells/mm^3^, with the majority having a CD4 count between 200 and 500 cells/mm^3^ (49.5%, 199/402), while the median viral load was 4,510 (838-8,780) copies/ml, with approximately half of the participants having a viral load of 1,000-10,000 copies/ml (52.2%, 210/402) (Table 2).

**Table 2:** Socio-biological characteristics of the study participants. N: total population; IQR: interquartile range; OCP: oral contraceptive pill. The data have been represented as frequency (N), percentage (%), and median (IQR).

Variables	Frequency N=402	Percentage (%)
Parity		
0	63	15.7
1	68	16.9
≥2	271	67.4
Median (IQR)	4 (2-5)	
Number of lifetime sexual partners		
1-5	23	5.7
6-10	367	91.3
>10	12	3.0
Median (IQR)	8 (7-9)	
History of multiple sexual partners in spouse		
Yes	389	96.8
No	13	3.2
Use of condom		
Always	87	21.6
Occasionally	209	52.0
Never	106	26.4
Ever used OCP		
Yes	50	12.4
No	352	87.6
History of alcohol consumption		
Regularly	107	26.6
Occasionally	227	56.5
Never	68	16.9
History of smoking		
Yes	23	5.7
No	379	94.3
CD4 counts (cells/mm^3^)		
<200	40	10.0
200–500	199	49.5
>500	163	40.5
Median (IQR)	440 (291-593)	
Viral load (copies/ml)		
<1,000	120	29.9
1,000 – 10,000	210	52.2
>10,000	72	17.9
Median (IQR)	4510 (838 – 8,780)	

Prevalence and distribution of AGWs

**Figure 1:**
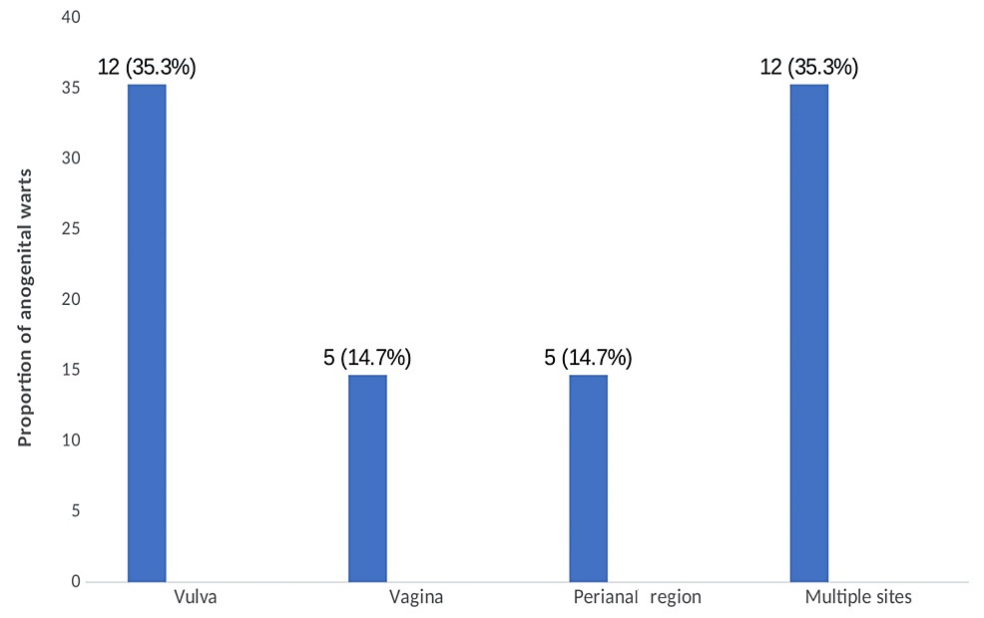
Site distribution of AGWs among recently diagnosed HPW on antiretroviral drugs (n=34). AGWs: anogenital warts; HPW: HIV-positive women. The data have been represented as frequency (n) and percentage (%).

The prevalence of AGWs among recently diagnosed HPW was 8.5% (34/402). The majority of the warts were located on the labia (35.3%, 12/34), followed by the vagina (14.7%, 5/34) and perianal region (14.7%, 5/34), while 35.3% (12/34) were located on more than one region, commonly the labia and perianal regions (Figure 1).

Demographic and socio-biological factors associated with AGWs among HPW

It was observed that occupation (p=0.005), marital status (p<0.001), and educational status (p=0.028) were the only demographic factors that were significantly associated with the occurrence of AGWs among HPW. The majority of the HPW who had AGWs were unemployed (32.4%, 11/34), single (47.1%, 16/34), and did not have tertiary education (94.1%, 32/34). More than two-thirds of the AGWs occurred in women between the ages of 20 and 39 years, with women <20 years and ≥40 years having the lowest prevalence of AGWs. However, age was not significantly associated with the presence of AGWs (p=0.312) (Table 3).

**Table 3:** Demographic factors associated with AGWs among study participants. AGWs: anogenital warts. p-value <0.05 is considered significant. The data have been represented as frequency (n) and percentage (%). *Fisher's exact test.

Variables	AGWs			p-value
	Present, n=34 (%)	Absent, n=368 (%)	Total, N=402 (%)	
Age groups (in years)				0.312*
<20	4 (11.8)	19 (5.2)	23 (5.7)	
20–29	15 (44.1)	147 (39.9)	162 (40.3)	
30–39	11 (32.4)	163 (44.3)	174 (43.3)	
≥40	4 (11.8)	39 (10.6)	43 (10.7)	
Occupation				0.005*
Unemployed	11 (32.4)	42 (11.4)	53 (13.2)	
Unskilled labor	10 (29.4)	170 (46.2)	180 (44.8)	
Skilled labor	10 (29.4)	132 (35.9)	142 (35.3)	
Professionals	3 (8.8)	24 (6.5)	27 (6.7)	
Marital status				<0.001*
Single	16 (47.1)	99 (26.9)	115 (28.6)	
Married	12 (35.3)	246 (66.8)	258 (64.2)	
Divorced	3 (8.8)	18 (4.9)	21 (5.2)	
Widowed	3 (8.8)	5 (1.4)	8 (2.0)	
Educational status				
No formal education	11 (32.4)	51 (13.9)	62 (15.4)	0.028*
Primary education	6 (17.6)	105 (28.5)	111 (27.6)	
Secondary education	15 (44.1)	170 (46.2)	185 (46.1)	
Tertiary education	2 (5.9)	42 (11.4)	44 (10.9)	

Some socio-biological factors were found to be associated with the occurrence of AGW. The use of OCP and smoking were significantly associated with HPW having AGW (p<0.001, respectively). Similarly, lower levels of CD4 count and higher levels of viral load were significantly associated with the occurrence of AGW among HPW (p<0.001, respectively). On the other hand, parity, total number of lifetime sexual partners, use of condoms, and alcohol intake were not significantly associated with having AGW (p>0.05) (Table 4).

**Table 4:** Socio-biological factors associated with AGWs among study participants. AGWs: anogenital warts; OCP: oral contraceptive pill; IQR: interquartile range. p-value <0.05 is considered significant. The data have been represented as frequency (n), percentage (%), and median (IQR). *Fisher's exact test. ^#^Pearson's chi-square test.

Socio-biological variables	AGWs			p-value
	Present, n=34 (%)	Absent, n=368 (%)	Total, N=402 (%)	
Parity				0.060^#^
0	10 (29.4)	53 (14.4)	63 (15.7)	
1	6 (17.6)	62 (16.8)	68 (16.9)	
≥2	18 (52.9)	253 (68.8)	271 (67.4)	
Number of lifetime sexual partners				0.060*
1–5	1 (2.9)	22 (6.0)	23 (5.7)	
6–10	31 (91.2)	336 (91.3)	367 (91.3)	
>10	2 (5.9)	10 (2.7)	12 (3.0)	
Usage of condom				0.161*
Always	3 (8.8)	84 (22.8)	87 (21.6)	
Occasionally	20 (58.8)	189 (51.4)	209 (52.0)	
Never	11 (32.4)	95 (25.8)	106 (26.4)	
Usage of OCP				<0.001^#^
Yes	12 (35.3)	38 (10.3)	50 (12.4)	
No	22 (64.7)	330 (89.7)	352 (87.6)	
Alcohol intake				0.421*
Regular intake	10 (29.4)	97 (26.4)	107 (26.6)	
Occasional intake	21 (61.8)	206 (55.9)	227 (56.5)	
Never	3 (8.8)	65 (17.7)	68 (16.9)	
Smoking				<0.001*
Yes	20 (58.8)	3 (0.8)	23 (5.7)	
No	14 (41.2)	365 (99.2)	379 (94.3)	
CD4 (cell/mm^3^)				<0.001*
<200	31 (91.2)	9 (2.5)	40 (10.0)	
200–500	2 (5.9)	197 (53.5)	199 (49.5)	
>500	1 (2.9)	162 (44.0)	163 (40.5)	
Median (IQR)	101 (55-164)	470 (325-934)		
Viral load (copies/ml)				<0.001*
<1,000	0 (0.0)	120 (32.6)	120 (29.9)	
1,000–10,000	10 (29.4)	200 (54.3)	210 (52.2)	
>10,000	24 (70.6)	48 (13.1)	72 (17.9)	
Median (IQR)	67,000 (8,650-132,714)	3,880 (764-8,020)		

## Discussion

This study aimed to investigate the prevalence, pattern, and epidemiological factors related to clinically diagnosed AGWs among recently diagnosed HPW who were receiving ART. The prevalence of AGWs was found to be 8.5% (34/402), with the majority of lesions observed on the labia. Occupation, marital status, and educational status were the significant demographic factors associated with AGWs among HPW. Additionally, sociobiological factors such as the use of OCPs, smoking habits, CD4 count, and viral load were significantly associated with AGW.

HIV infection is commonly recognized as a substantial risk factor for the development of AGWs, with a higher prevalence and incidence observed among individuals living with HIV and AIDS compared to those who are not infected [17,20]. This is consistent with the findings in our study where the prevalence of AGWs (8.5%, 34/402) among HPW is higher than that reported among non-HIV-infected women by Dareng et al. (1.0%) [9] in Nigeria and Low et al. (1.6%) [21] in Burkina Faso among non-HIV-infected women. A systematic review and meta-analysis reported that the prevalence of AGW was five times higher in HPW compared to women who are HIV-negative [17]. This finding can be attributed to the increased susceptibility of HPW to multiple HPV infections, and compromised cell-mediated immunity, which hinders the clearance of the HPV infection in this population [9,22].

There is a lack of available data regarding the prevalence and epidemiological characteristics of AGW among HPW in our environment. Our study revealed a higher prevalence of AGW compared to the findings reported by another author in Abuja, Nigeria. In Abuja, the prevalence rate was documented to be 5.0% [9]. These findings align with research conducted in Russia, which indicates that the prevalence of AGW among women varies across different regions of the country, ranging from 4.9% to 8.9% [19]. The prevalence observed in our study was higher than the reported figure in some other African countries like South Africa (5.7%) and Burkina Faso (7.0-7.5%) [13,21]. This is not unexpected, as the prevalence of AGW is influenced by various factors such as geographical location, socio-cultural characteristics, sexual behavior, the prevalence of HIV infection, and immune susceptibility within the studied population [9,21].

The study revealed that demographic factors, including occupation, educational level, and marital status, exhibited a significant association with the presence of AGW in HPW. The prevalence of AGW was notably higher among women who were unemployed and had a lower level of education in the HPW population. Occupation and educational attainment significantly influence the socioeconomic status of individuals. A correlation has been consistently observed between low level of education, occupation, and low socioeconomic status, which has been identified as a risk factor for having AGW [9]. According to the available data, individuals with higher levels of education or occupations in professional fields tend to exhibit a reduced likelihood of experiencing AGW [21]. However, it is essential to note that these findings contradict the results of another study, where education and occupation did not impact the risk of developing AGW [14].

Marital status was associated with AGWs among HPW in our study. HPW who are single were more likely to have AGWs compared to those who were married. This observation aligns with the results reported in certain studies, yet it contradicts the findings reported in another study [9,14]. This phenomenon can likely be attributed to the increased vulnerability of unmarried women to engaging in risky sexual behaviors, such as having multiple sexual partners, engaging with partners who have multiple sexual partners, and having a high number of sexual partners [23-25], which are risk factors for AGW. Although the majority of women in our study who had AGW were under the age of 30, our analysis did not find a significant association between age and the diagnosis of AGW. These findings align with the results observed in certain studies [21,25] and are contrary to others [9,23].

The use of hormonal contraceptives, particularly OCPs, has been found to be linked to the potential risk of developing AGWs [23,26-28]. This is consistent with the findings in our study, where a significant proportion of HPW with AGWs had a history of OCP use compared to those without AGWs. Conversely, OCP was not associated with an increased risk of AGW in some other studies [29]. It is believed that steroids such as estrogen and progesterone can enhance the transcription of HPV and suppress both cellular and non-cellular immune responses to HPV infection [26]. This probably underlines the role of OCP in the persistence of HPV infection.

Similar to findings from our study, smoking has been shown in several studies to be associated with an increased risk of AGW in women and men irrespective of their HIV status [17,21,27,28]. This risk is probably dose-dependent, with heavy smokers having an increased risk compared to non-heavy smokers [1,30]. Immune suppression induced by smoking has been suggested as a possible reason for this association [1,30].

Research has demonstrated that sexual behavior is a significant risk factor linked to the diagnosis of AGWs in both men and women. This suggests that AGW is acquired through sexual transmission [30]. Studies have documented a positive correlation between an increased number of lifetime sexual partners and the risk of developing AGWs [23,27,29,30], but this was not associated with having AGWs in our studies. This is probably due to the generally high rate of sexual activities and a high number of lifetime sexual partners reported among the study participants, irrespective of the diagnosis of AGW.

PLWHA are typically more susceptible to AGWs compared with the non-HIV-positive population due to the higher risk of acquisition and persistence of HPV infection from immunosuppression. Viral load (VL) and CD4 count levels are surrogate markers of the severity of HIV infection and immune suppression among PLWHA. Several studies have shown a significant correlation between the level of VL, CD4 count, and risk of developing AGWs among PLWHA [3,4,9,11,13-15,17,20,21]. High levels of VL and low CD4 count levels are associated with AGWs in this group of people, which is consistent with findings from our study. This buttresses the need for early detection and treatment of HIV infection and the importance of HPV vaccination among this population to prevent HPV-related diseases like AGW.

Limitations

Like in any study, our study has its limitations. The diagnosis of AGWs in this study was made clinically by inspecting the anogenital region. Though histological diagnosis is the most reliable way to diagnose AGW, most AGWs are usually diagnosed clinically. Findings from this study may not reflect the factual findings in the general population of HPW as it is a hospital-based study, and it only focused on a subset of recently diagnosed HPW on ART. Since all the participants were on ART and the duration of the use of ART was relatively short, the study assumed that the use of ART had no impact on the occurrence of AGW.

## Conclusions

Based on our findings, we conclude that the prevalence of AGW among recently diagnosed HPW was 8.5% (34/402), with the majority of AGW located on either the labia majora or minora. Demographic factors such as occupation, marital status, and educational status were significantly associated with a diagnosis of AGW, with women who were unemployed, single, and without tertiary education having a higher prevalence of AGW. Similarly, low CD4 count level, high viral load, history of the use of OCP, and smoking were socio-biological factors associated with the diagnosis of AGW among HPW. There is a need to further evaluate the impact of these epidemiological factors in more robust studies.
